# Single nucleotide polymorphism profiles of canine T-cell and null-cell lymphomas

**DOI:** 10.3389/fvets.2024.1439706

**Published:** 2024-08-07

**Authors:** Sirintra Sirivisoot, Tanit Kasantikul, Somporn Techangamsuwan, Anudep Rungsipipat

**Affiliations:** ^1^Center of Excellence for Companion Animal Cancer, Department of Pathology, Faculty of Veterinary Science, Chulalongkorn University, Bangkok, Thailand; ^2^Veterinary Diagnostic Laboratory, Michigan State University, Lansing, MI, United States

**Keywords:** cutaneous T-cell lymphoma, dog, intestinal T-cell lymphoma, nodal T-cell lymphoma, null-cell lymphoma, SNPs

## Abstract

**Background:**

The histopathological classification of T-cell lymphoma (TCL) in humans has distinctive mutational genotyping that suggests different lymphomagenesis. A similar concept is assumed to be observed in dogs with different TCL phenotypes.

**Objective:**

This study aimed to identify the previously reported single-nucleotide polymorphisms (SNPs) in both human beings and dogs in canine TCLs and null-cell lymphomas (NCLs) and to design compatible oligonucleotides from each variant based on the multiplex polymerase chain reaction.

**Methods:**

Genomic DNA was extracted from 68 tumor specimens (62 TCLs and 6 NCLs) and 5 buffy coat samples from dogs with TCL. Four TCL subtypes and NCL were analyzed in 44 SNPs from 21 genes using the MassARRAY.

**Results:**

The greatest incidences of SNPs observed in all TCL subtypes and NCL ware *SATB1* c.1259A > C, *KIT* c.1275A > G, *SEL1L* c.2040 + 200C > G, and *TP53* c.1024C > T, respectively. Some SNP locations were statistically significant associated with NCL, including *MYC* p.S75F (*p* = 0.0003), *TP53* p.I149N (*p* = 0.030), *PDCD1* p.F37LX (*p* = 0.012), and *POT1* p.R583* (*p* = 0.012).

**Conclusion:**

Each TCL histological subtype and NCL are likely to contain distinctive mutational genetic profiles, which might play a role in lymphoma gene-risk factors and might be useful for selecting therapeutic target drugs for each canine patient.

## Introduction

Canine lymphoma can be categorized into B-cell, T-cell, and null-cell (non-B, non-T-cell) lymphomas (1). Regardless of the prevalence of TCL and rare NCLs, a frequent histopathological subtype of TCL is peripheral TCL, not otherwise specified (PTCL-NOS) (2). Other less common subtypes include T-zone lymphoma (TZL), enteropathy-associated T-cell lymphoma (EATCL), and cutaneous T-cell lymphoma (CTCL) (3). CTCL can be either epitheliotropic (ECTCL) or non-epitheliotropic (NECTCL). Canine TCL often has a worse prognosis than B-cell lymphoma (4); however, each TCL subtype often shows a variable prognosis and disease outcome. TZL has a better prognosis because of its indolent disease course (5). Based on the anatomic locations of TCL, patients with hepatosplenic and gastrointestinal lymphoma often have shorter survival times than those with certain forms of multicentric or cutaneous lymphoma (6–10). Moreover, the overall survival time of dogs with ECTCL is shorter than that of dogs with NECTCL (11). Due to their differences in prognosis, a unique therapy for each TCL subtype may be required. Many studies have suggested multidrug chemotherapy for TCL, such as a combination of mechlorethamine, vincristine, prednisone, and procarbazine (12); lomustine, vincristine, procarbazine, and prednisolone (13), or vincristine, L-asparaginase, doxorubicin, cyclophosphamide, actinomycin-D, procarbazine, prednisolone, and lomustine (14), to improve progression-free survival and overall survival time in lymphoma dogs. However, CHOP-based induction (vincristine, cyclophosphamide, doxorubicin, and prednisolone) is still preferred by veterinary oncologists, and lomustine-based protocols are used as rescue treatments (15).

Specific molecular signatures of PTCL-NOS in humans can be classified into *TBX21* and *GATA3* subgroups, which are T helpers (T_H_)1 and T_H_2-cell differentiation regulators, respectively (16). PTCL-NOS-*GATA3* frequently exhibited mutations of *PTEN* and *TP53*, with co-occurring amplifications of *MYC* and *STAT3*. For PTCL-NOS-*TBX21*, mutations of DNA methylation regulator genes were noted in *TET1*, *TET3,* and *DNMT3A* (17). For human CTCL, specific genetic mutations were involved in T-cell activation (*CD28* and *RHOA*), cell apoptosis (*FAS*), NF-κB pathways (*NFKB2* and *STAT5B*), chromatin remodeling (*DNMT3A* and *ARD1A*), and DNA damage response (*TP53* and *CDKN2A*) (18). Moreover, the loss-of-function mutation of *PDCD1*—the gene encoding an inhibitory receptor program death protein 1 (PD-1)—was driven by aggressive behavior in CTCL (19). For EATCL, the most frequently mutated genes were associated with the JAK–STAT pathway in *SETD2*, *STAT5B*, *JAK1*, *JAK3*, and *STAT3* (20–22). Hence, these findings are useful for selective potential therapeutic opportunities in each TCL subtype as a single agent or in combination with anti-neoplastic drugs. For example, the NF-κB inhibitor bortezomib and the PI3K-δ,λ inhibitor duvelisib were applied in clinical trials treating PTCL and CTCL, and the overall response rate ranged from 31 to 67% (23, 24).

In canine TCLs, Labadie et al. reported SNPs in TZL using genome-wide association mapping (25). Several SNPs of hyaluronidase genes were associated with disease risk for TZL, including *SPAM1*^K482R^, *HYALP1*^M463T^, and *HYAL4*^G454S, S434F, L378I^. Another study investigated somatic mutations in a variable group of TCL with limited subtyping in Golden retrievers and Boxers using whole-exome sequencing (26). The common mutated genes in both breeds were noted on *SATB1* c.1259 A > G p.Q420R, while *PTEN* c.975 C > T p.L325 = was found in Boxer-TCLs (26, 27). Similar variants using RNA sequencing were found in two dogs with PTCL (27). Three somatic missense mutations were described in canine TCL, including *MYC* c.185C > T p.Ser62Phe, *TP53* c.715G > A p.Arg239Trp, and *MET* c.3804C > G p.Asp1268Glu (28). Nonetheless, no study has examined novel SNPs associated with human TCL in dogs. Thus, our study aimed to investigate SNP genotyping patterns in canine PTCL, ECTCL, NECTCL, EATCL, and NCL based on the important gene-related lymphoma risks in human and canine TCLs (19, 29–31) and to obligate primer design for multiplex PCR, using the MassARRAY platform (Agena Bioscience, CA, United States).

## Materials and methods

### Study samples and immunohistochemistry

Formalin-fixed paraffin-embedded (FFPE) block archives were retrieved from the Department of Pathology, Faculty of Veterinary Science, Chulalongkorn University, between 2008 and 2021 and from three private veterinary labs (SQ Reference Lab, China; Vet Central Lab, Thailand; and Vet Clinical Center, Thailand) between 2020 and 2021. Only patients who had not previously received chemotherapeutic drugs were enrolled in this study. According to physical examination, abdominal ultrasonography, and buffy-coated smears, all lymphoma dogs were in at least WHO clinical stage III (15). The selected cases included multicentric, cutaneous, and alimentary lymphomas that were recut into 3-μm thickness, stained with hematoxylin and eosin, and immunostained against CD3 (Peter F. Moore, United States), CD20 (ab27093, Abcam, United States), and CD79a (HM57, Abcam) or Pax5 (1EW, Leica, United Kingdom). Immunohistochemistry was performed as described elsewhere (30). Only lymphoma samples that had no immunoreactivity for B- and T-cell markers were further investigated with CD18 (histiocyte marker, Peter F. Moore), CD117 (mast cell marker, MIB1, Dako), and MUM1 (plasma cell marker, ab133590, Abcam) to rule out the possibility of other hematopoietic lineages. Finally, cases that showed negative to specific leucocyte markers but positively immunostained with CD45 (common leucocyte antigen) were assigned as NCL.

To evaluate the effects of missense *SATB1*^Q420P^ mutation on programmed death ligand 1 (PD-L1) and missense *TP53*^I149N^ mutation on p53, five samples from each group (four *SATB1* mutants and one *SATB1* wild type vs. two *TP53* mutants and three *TP53* wild types) were immunolabeled against PD-L1 and p53, respectively. In brief, the tissue section was preheated by citrate buffer pH 6 at 95°C for 20 min and blocked endogenous peroxidase and non-specific binding protein by 0.3% (v/v) H_2_O_2_ for 30 min and 5% (w/v) bovine serum albumin for 20 min, respectively. Anti-CD45 antibody (Peter F. Moore) was incubated in a 1:10 dilution at room temperature (RT) for 1.5 h, while PD-L1 (1:100, clone 5D2) (32) and p53 (1: 500, PA5-27822, Invitrogen, United States) were incubated at RT for 2 h. Envision mouse/rabbit-HRP (Dako, Hilden, Germany) was used as a secondary antibody, and 3,3′-diaminobenzidine was used as a substrate. Histopathological diagnosis was made by a board-certified veterinary pathologist (TK) according to WHO classifications (3). Signalment and clinical history of all selected cases were recorded; however, most cases were lost due to a lack of treatment follow-up.

### DNA extraction

A total of 70–100 μm FFPE tissue scrolls were collected into a sterile 1.5 mL microtube. The genomic DNA from each sample was then extracted using a DNeasy Blood and Tissue Kit (Qiagen, Germany). After deparaffinization with xylene and absolute ethanol, the sample was processed according to the manufacturer’s instructions. The DNA concentration of each sample was then measured with a NanoDrop Lite Spectrophotometer (Thermo Scientific, United States). The requirement of high-quality DNA for targeted SNP genotyping using the Agena Bioscience MassARRAY system is a 260/280 ratio of >1.8 and a concentration of >10 ng/μL.

### iPLEX genotyping process

The dog reference genomes were obtained from Dog10K_Boxer_Tasha and UMICH_Zoey_3.1.^1^ MassARRAY was used for genotyping of the 44 targeted SNPs in 21 different genes, as shown in Table 1. The SNP locations were selected based on the previous studies (19, 26, 27, 29–31, 36). A primer pair for each location was designed by AgenaCx software (Agena Bioscience). First, multiplex PCR primers were amplified in the target regions by using a 5-uL reaction of 0.5 μL of 10X PCR buffer (Agena Bioscience), 2 mM MgCl_2_, 500 μM dNTPs, 0.5 μM of each amplification primer, and 0.2 U DNA polymerase. Second, 1.7 U shrimp alkaline phosphatase (SAP, Agena Bioscience) and 0.17 μL of 10X SAP buffer were added to the first-step PCR product to dephosphorylate residual nucleotides. Third, the iPLEX extension reaction was composed of 0.2 μL of 10X iPLEX buffer (Agena Bioscience), 0.2 μL of 10X dNTP/ddNTP combination, 0.142 U iPLEX enzyme, and 0.5–1.57 μM extend primer mix. These three steps were carried out by T100 thermal cycler (Bio-Rad, United States), and the cycling conditions were followed by Sirivisoot et al. (30). The final extension products were desalted and transferred onto a SpectroChip with an automated nano-dispenser. The different sizes of SNP variations were identified using a matrix-assisted laser desorption/ionization time-of-flight mass spectrometer. Genotyping of each target site was presented as mass spectrum peaks using a MassARRAY analyzer. The percentage of determinate 44 variants (%call rate) for each sample must be achieved by ≥90%.

**Table 1:** A total of 44 specified single nucleotide polymorphisms (SNPs) were selected and investigated in canine T-cell and null-cell lymphomas.

Genes	SNPs	Locations	Mutations	Existing variants	References
*KIT*	c.1275A > G	13:47131711	Thr425=	rs22299980	(30, 33)
*PTEN*	c.975C > T	26:37346711	Leu325=	–	(26, 27, 30)
*ENSCAFG00000024436* (*HYALP6*)	c.1317A > G	14:11444847	Leu439=	–	(25, 30)
*LMNB1*	c.1184C > T	11:14831257	Ser395Leu	–	(30, 34)
*MET*	c.3804C > G	14:55094583	Asp1268Glu	–	(28, 30)
*MVB12A*	c.361G > A	20:45281784	Asp121Asn	–	(30, 34)
*MYC*	c.224C > T	13:25171460	Ser75Phe	–	(28, 30)
*SATB1*	c.1259A > C	23:24686804	Gln420Pro	–	(26, 27, 30)
*TP53*	c.709C > T	5:32702350	Arg237Trp	rs852661628	(28–30)
	c.1024C > T	5:32701110	Gln342Ter	–	
	c.311_312insA	5:32703552	Thr105AspfsTer47	–	
	c.446 T > A	5:32702916	Ile149Asn	–	
	c.640_641insT	5:32702418	Gly214ValfsTer3	–	
*PDCD1*	c.136G > T	25:51833128	Glu46Ter	–	(31)
	c.233A > G	25:51833031	Lys78Arg	–	
	c.108_109insCT	25:51833155	Phe37LeufsTer35	–	
*POT1*	c.850C > T	14:10733311	Arg284Cys	–	(30, 35)
	c.927del	14:10733387	Phe309LeufsTer3	–	
	c.1747C > T	14:10752708	Arg583Ter	–	
	c.1928 T > C	14:10756216	Phe643Ser	–	
*TRAF3*	c.850A > T	8:70295454	Lys284Ter	rs851689319	(30, 35)
	c.906del	8:70295509	Ile302MetfsTer21	–	
	c.908dup	8:70295512	Arg304GlufsTer9	–	
	c.942_949dup	8:70295553	Leu317ProfsTer9	–	
	c.968_971del	8:70300522	Ile323ThrfsTer7	–	
	c.1652del	8:70302097	Asp551ValfsTer9	–	
	c.1434_1445del	8:70301879	Met478_Tyr482delinsIle	–	
	c.1591_1592insTC	8:70302037	Ala531ValfsTer14	–	
	c.1339del	8:70301784	Thr447ArgfsTer14	–	
	c.1195del	8:70301640	Leu399TrpfsTer20	–	
*STAT3*	c.1919A > T	9:20069778	Tyr640Phe	–	(19)
*RHOA*	c.350A > T	20:39804089	Asn117Ile	–	(19)
	c.351C > G	20:39804090	Asn117Lys	–	
	c.351C > A		Asn117Lys	–	
*SPAM1*	c.1445A > T	14:11388961	Lys482Met	–	(25, 30)
	c.1445A > G		Lys482Arg	rs851582160	
	c.1445A > C		Lys482Thr	–	
*FLT3*	c.10 + 1601A > C	25:11645948	Intron variant	–	(30, 33)
	c.10 + 1830A > G	25:11646177	Intron variant	–	
	c.10 + 13857G > C	25:11658204	Intron variant	–	
*ZNHIT6*	c.-14G > C	6:64984982	5’UTR variant	–	(30, 34)
*DIO2*	c.-128-3748 T > G	8:52321507	Intron variant	–	(25, 30)
*SEL1L*	c.2040 + 200C > G	8:53336337	Intron variant	–	
	c.1248 + 56G > C	8:53344119	Intron variant	–	
	c.777 + 1,097 T > C	8:53354569	Intron variant	–	
*SNORD3A*	G > A	14:11463068	Upstream gene variant	–	
*ENSCAFG00000053717*	G > A	14:11491220	Upstream gene variant	–	

### Statistical analysis

A total of 44 SNP locations were calculated for the correlation with anatomical forms and histological subtypes of TCL/NCL using the chi-square test by GraphPad Prism version 9.0 for macOS (GraphPad Software, United States).

## Results

### Patient information

The demographics of the 68 dogs are shown in Table 2. The median age of dogs with TCL/NCL was 8 years (ranging from 1 to 15 years). Among the 35 purebred dogs, the most prevalent breeds were Golden retriever (28.57%, 10/35), Shih Tzu (14.29%, 5/35), Poodle (8.57%, 3/35), and Labrador retriever (8.57%, 3/35).

**Table 2:** Demographic information of 68 dogs with each T-cell lymphoma subtype and null-cell lymphoma.

	PTCL (*n* = 18)	ECTCL (*n* = 21)	NECTCL (*n* = 15)	EATCL (*n* = 8)	NCL (*n* = 6)
Dog breed					
Purebred	11	10	6	5	3
Mixed	3	6	4	–	3
Unknown	4	5	5	3	–
Gender					
Male	5	9	4	4	3
Mc	2	1	1	1	1
Female	5	3	4	1	1
Fs	2	4	2	–	1
Unknown	4	4	4	2	–
Mean age in years (age range)	6.8 (2–15)	8.1 (3–14)	6.6 (1–15)	10.5 (8–13)	9.2 (6–15)

EATCL, enteropathy-associated T-cell lymphoma; ECTCL, epitheliotropic cutaneous T-cell lymphoma; Fs, sprayed female; Mc, castrated male; NCL, null-cell lymphoma; NECTCL, non-epitheliotropic cutaneous T-cell lymphoma; PTCL, peripheral T-cell lymphoma.

**Figure 1:**
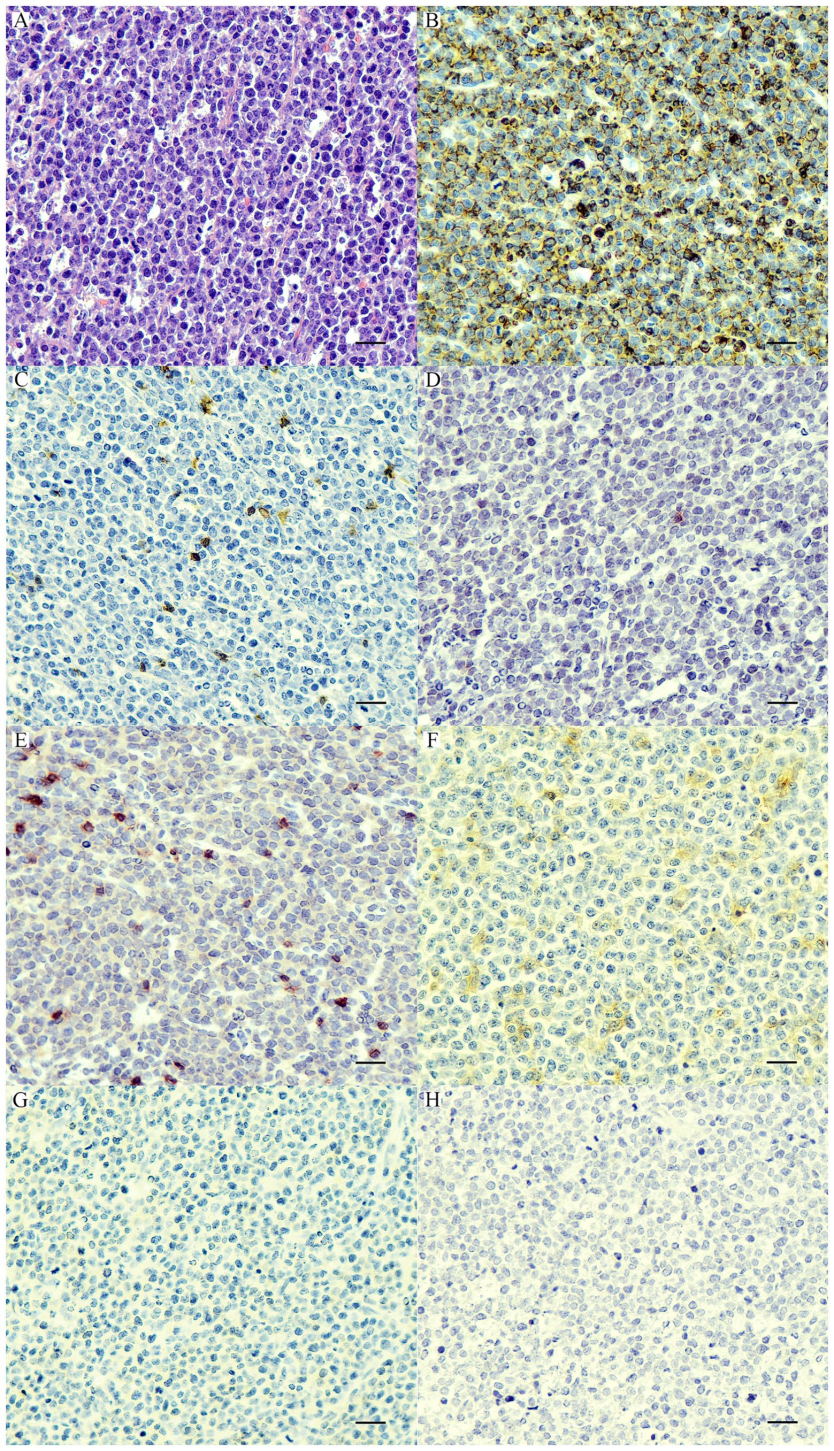
Nodal null-cell lymphoma. **(A)** The nuclei of neoplastic cells are large >2× of a red blood cell, finely stipple to vesiculate chromatin, and contain 1–2 prominent nucleoli with a scant amount of cytoplasm. H&E. Bar = 10 μm. The neoplastic cells show cytoplasmic immunolabeling with CD45 **(B)** and are negative for CD20 **(C)**, CD79a **(D)**, CD3 **(E)**, CD18 **(F)**, CD117 **(G)**, and MUM1 **(H)**. IHC. Bar = 10 μm.

Based on the anatomical locations, 21 cases were multicentric, 39 cases were cutaneous, and 8 cases were alimentary. According to histopathological and immunophenotyping results of 68 cases, there were 18 PTCLs, 21 ECTCLs, 15 NECTCLs, 8 EATCLs type I, and 6 cases of NCL (CD3-, CD20-, CD79a-, CD117-, CD18-, MUM1-, and C45+) (Figure 1). Among four aberrant TCL cases (CD3+, CD20+, and Pax5-), one dog had nodal PTCL, and three dogs had ECTCL. For two biphenotypic TCLs (CD3+, CD20+, and Pax5+), one case had nodal PTCL and the other case had EATCL.

### iPLEX genotyping results

**Figure 2:**
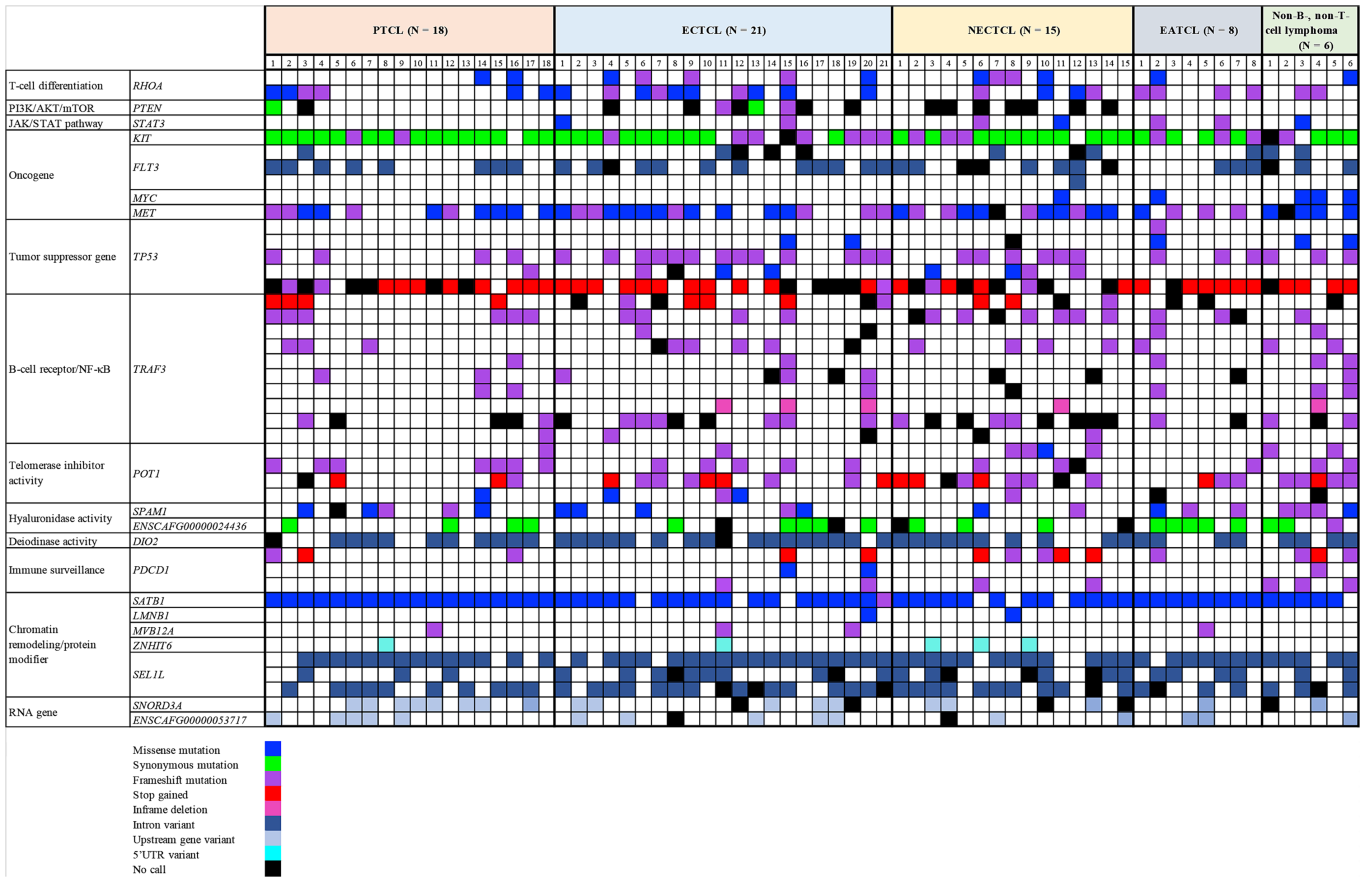
Landscape of single-nucleotide polymorphisms in canine T-cell and null-cell lymphomas. Genomic analysis of 21 mutated genes was harbored in 68 dogs with 4 T-cell lymphoma variants: peripheral T-cell lymphoma (PTCL), epitheliotropic cutaneous T-cell lymphoma (ECTCL), non-epitheliotropic cutaneous T-cell lymphoma (NECTCL), enteropathy-associated T-cell lymphoma (EATCL), and null-cell lymphoma (NCL). *No call indicates a failure to detect either a wild-type or mutant peak.

Each lymphoma dog had a different mutational genotyping profile, even though they had a similar TCL subtype (Figure 2). The highest genetic variations frequently observed in all TCL/NCL subtypes were *SATB1* c.1259A > C p.Gln420Pro (89.71%, 61/68), *KIT* rs22299980 p.Thr425 = (89.39%, 59/66), *SEL1L* c.2040 + 200C > G (82.35%, 56/68), and *TP53* c.1024C>T p.Gln342Ter (80.39%, 41/51). When comparing each SNP across anatomic locations, some SNP variants were frequently observed in specific forms, regardless of statistical significance. For instance, the SNPs mainly found in cutaneous TCL were *TP53* c.640_641insT (53.85%, *p* = 0.08). *SEL1L* c.1248 + 56G > C was noted in 50% of intestinal TCL (*p* = 0.16). *SNORD3A* 14:11463068 G > A was regularly mutated in nodal TCL (40%, *p* = 0.12).

**Figure 3:**
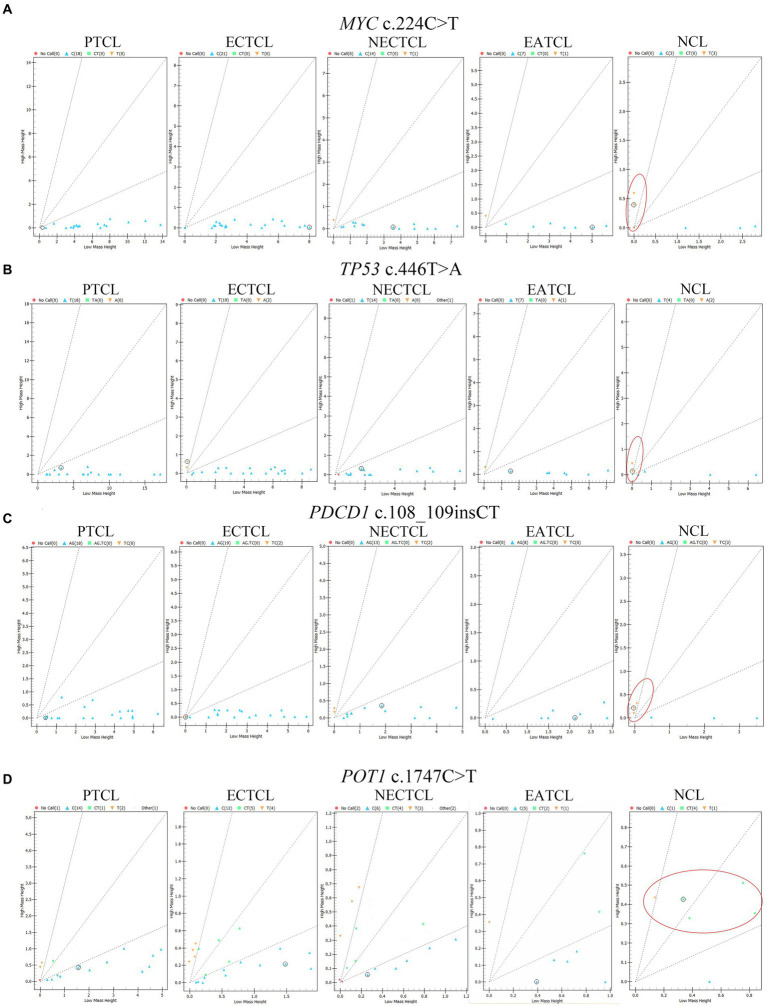
Cluster plots present mass spectra from each lymphoma dog in four single nucleotide polymorphisms significantly associated with the null-cell lymphoma subtype. **(A)** *MYC* c.224C > T (*p* = 0.0003), **(B)** *TP53* c.446 T > A (*p* = 0.03), **(C)** *PDCD1* c.108_109insCT (*p* = 0.012), and **(D)** *POT1* c.1747C > T (*p* = 0.012) were generally seen in NCL comparing to other subtypes (red oval). Blue triangle = wild type; green square; and yellow inverted triangle = mutant; red circle = no call; EATCL, enteropathy associated T-cell lymphoma; ECTCL, epitheliotropic cutaneous T-cell lymphoma; NCL, null-cell lymphoma; NECTCL, non-epitheliotropic cutaneous T-cell lymphoma; PTCL, peripheral T-cell lymphoma.

Each SNP location was analyzed to determine the association among five histological subtypes by chi-square test. *MYC* c.224C > T (p.Ser75Phe, *p* = 0.0003), *TP53* c.446 T > A (p.Ile149Asn, *p* = 0.030), *PDCD1* c.108_109insCT (p.Phe37LeufsTer35, *p* = 0.012), and *POT1* c.1747C > T (p.Arg583Ter, *p* = 0.012) were significantly mutated in NCLs (Figure 3).

All primer pairs of each SNP location had amplification efficiency in multiplex PCR of more than 90%, except for *TP53* c.1024C > T, *TRAF3* c.942_949dup (p.Leu317ProfsTer9), *PTEN* c.975C > T (p.Leu325=), and *TRAF3* rs851689319 (p.Lys284Ter), which had 75% (51/68), 79% (54/68), 81% (55/68), and 90% (61/68), respectively.

**Figure 4:**
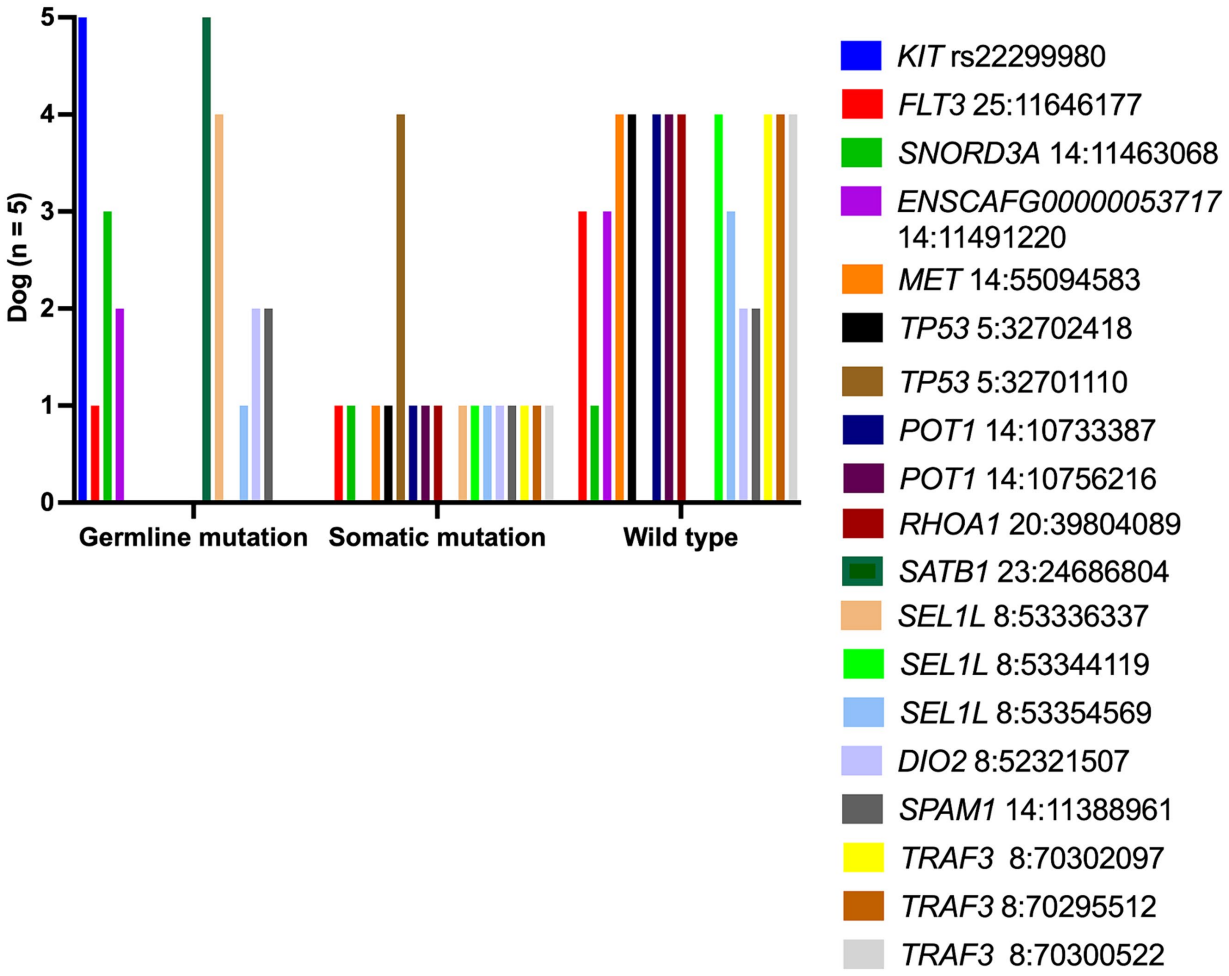
Bar chart illustrates SNP detection as germline or somatic in five canine TCLs.

To distinguish a germline from a somatic mutational profile, the authors selected five archival-matched buffy coat samples from PTCL dogs (dog no.7–10 and dog no.14) to assess this SNPs panel compared to TCL specimens. Somatic SNPs were uniquely varied in each dog, as shown in Figure 4. The frequent somatic mutation was seen in *TP53*^Q342*^(4 out of 4 dogs), while other somatic variants, for instance, *MET*, *TP53*, and *RHOA* were found in 20% (1 out of 5 dogs).

This study investigated the effect of *SATB1*^Q420P^ on PD-L1 expression in canine neoplastic T-cells. Four cases of mutant and one case of wild-type *SATB1* were immunolocalized for PD-L1. In four mutant dogs, neoplastic T lymphocytes expressed strong cytoplasmic intensity against PD-L1 compared to the wild type (Supplementary Figure S1). Therefore, T-cell anergy and exhaustion might be one of the lymphomageneses in dogs that are related to *SATB1* mutation.

Two NCLs having *TP53*^I149N^ were evaluated for their p53 expression status. Interestingly, both dogs with *TP53*^I149N^ had a loss of p53 expression (<1% of positive nuclei) when compared to three dogs with wild-typed *TP53*, which showed weak and heterogenous intensity of positive cells <10% (Supplementary Figure S2).

## Discussion

**Figure 5:**
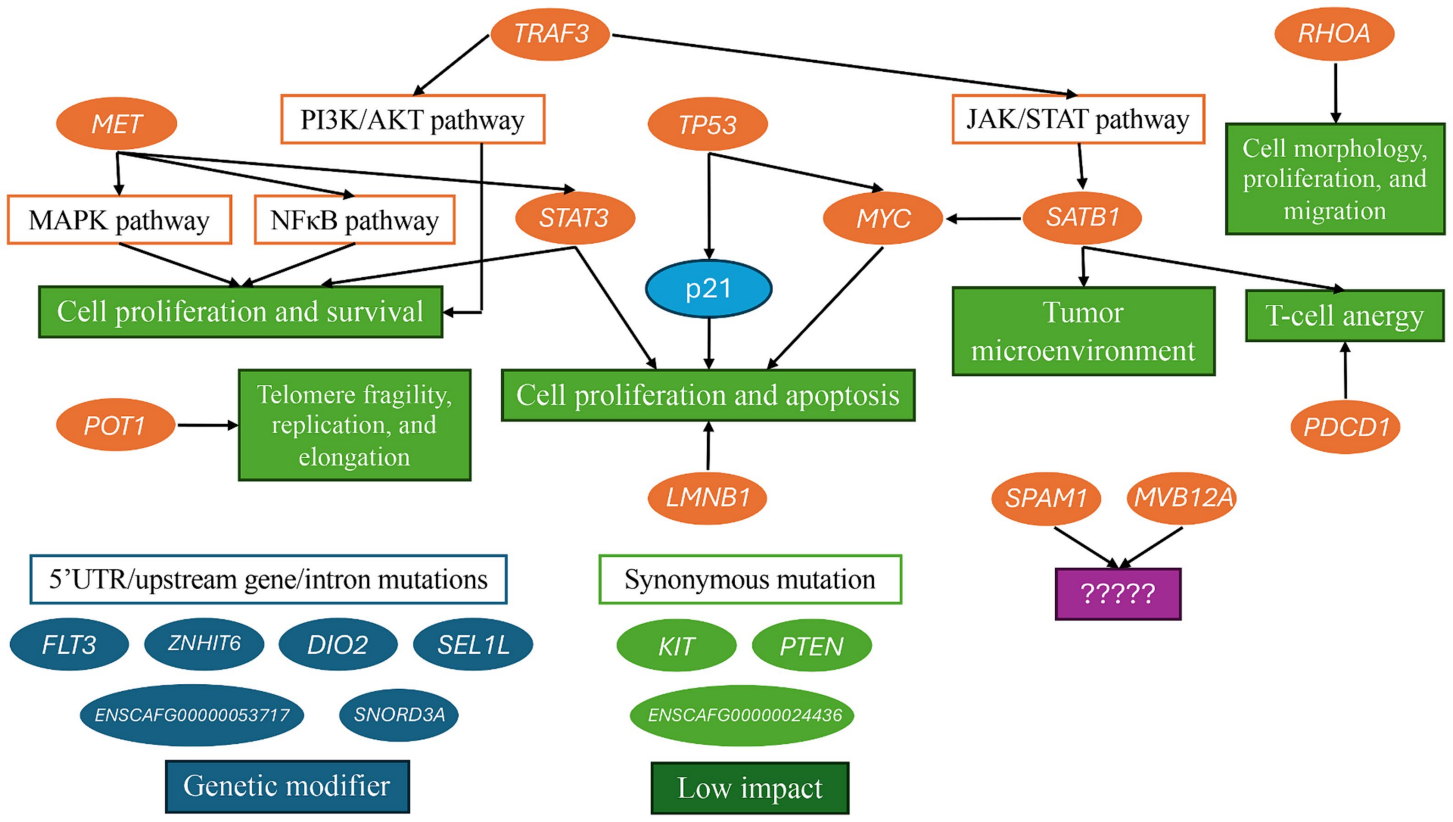
Potential pathways of 21 targeted genes contribute to lymphoma development.

In total, 44 targeted SNP panels were designed and evaluated using the MassARRAY in 68 FFPE specimens of canine TCLs and NCLs. The overall pathways of the targeted genes in this study are shown in Figure 5. Most variants were observed in TCL/NCL subtypes except for *MYC*^S75F^, *LMNB1*^S395L^, or *STAT3*^Y640F^ which were absent in PTCL. The highest frequency of genetic mutations in canine TCL was found at *SATB1*^Q420P^ and *KIT*^T425=^, respectively, and similar locations were reported in canine nodal B-cell lymphomas (30). However, the significance of the *KIT* mutation was synonymous, causing a low impact on carcinogenesis. The variants in hyaluronidase (*SPAM1* and *ENSCAFG00000024436/HYALP6*) and thyroid hormone regulation (*DIO2*) genes reported in canine TZL (25) were also detected in other histologic TCLs, including PTCL, ECTCL, NECTCL, EATCL, and NCL. Low molecular weight hyaluronan (a byproduct of ligand activation) has pro-inflammatory and pro-oncogenic effects that might be associated with cell proliferation, angiogenesis, and metastasis (37, 38). Moreover, three intron variants of *SEL1L* were present in 34–82% of the TCL/NCL subtypes. Intron mutations impact as a genetic modifier. *SEL1L* is an unfolded protein response gene that is stimulated during the accumulation of unfolded and misfolded proteins in endoplasmic reticulum stress and plays a role in the protein degradation pathway through the ubiquitin-proteosome system (39). In one study, downregulation of *SEL1L* significantly decreased the expression of *TIMP* and *PTEN* involving tumor invasion in human pancreatic cancer (40). Hence, the effect pathway of *SEL1L*, the mutation function of hyaluronidase genes, and hyaluronan expression levels need further investigation to confirm their contribution to lymphoma pathogenesis in dogs.

Mutations of *PTEN* and *SATB1* were frequently found in 12–25% of canine TCL (26, 27). In a recent study, a missense mutation of *SATB1*^Q420P^ was noted in TCL and NCL, whereas a synonymous mutation of *PTEN*^L325=^ was exhibited in one PTCL (5%), three ECTCLs (18%), and one EATCL (12%). *SATB1*, a global chromatin organizer, is dysregulated in human cutaneous TCL by promoting the expression of T_H_-2 cytokines (IL-5 and IL-9), which were appropriate for the tumor microenvironment (41). *SATB1* also demonstrated an inhibitory effect on *PD-L1* expression in T lymphocytes (42). A low *SATB1* mRNA expression level was related to an unfavorable prognosis in human mycosis fungoides and revealed decreased eosinophil infiltration, increased large-cell transformation, a high Ki-67 index, and elevated PD-1 expression (43). The SNPs of *MYC*^S75F^, *MET* (c.3804C > G, p.Asp1268Glu), and *TP53* (c.709C > T, p.Arg237Trp; c.1024C > T, p.Gln342Ter; c.311_312insA, p.Thr105AspfsTer47; c.446 T > A, p.Ile149Asn; and c.640_641insT, p.Gly214ValfsTer3) from previous publications (19, 28, 29) were also observed in the current study. *TP53*^Q342*^ (80.39%) and *MET*^D1268E^ (69.7%) were highly mutated in canine TCLs/NCLs. The oncogenic *MET* gene was upregulated in human PTCL and EATCL, and its gain-of-function contributed to T-cell lymphomagenesis (44, 45); therefore, it may play an important role in dogs as well.

Even though *POT1* and *TRAF3* polymorphisms were not described in four cases of canine TCL (35), we included them in our genotyping panel due to their detection in preliminary samples. A total of 4 variants of *POT1* and 10 variants of *TRAF3* were identified in 7–41% and 4–29% of all TCL/NCL specimens, respectively. A tumor necrosis factor receptor-associated factor 3 (*TRAF3*) serves as a tumor suppressor in B-cell lymphoma; nevertheless, it is required for the cell proliferation of anaplastic large T-cell lymphoma by activating the PI3K/AKT and JAK/STAT pathways (46). Another study of mutations affecting the protection of telomere 1 (*POT1*) explained that *POT1* inhibition in CTCL induced telomere fragility, replication fork stalling, and telomere elongation, which led to defective telomere replication during tumorigenesis (47). Thus, a significant mechanism of lymphoma development in dogs caused by *TRAF3* and *POT1* mutations pivotally requires further study.

Some SNP locations in *RHOA*, *STAT3*, and *PDCD1* were frequently described in human TCLs; however, no study has investigated them in dogs with TCL. *RHOA* encodes a small GTPase that switches signal transduction cascades and promotes cytoskeleton organization, cell migration, and the cell cycle (48). The missense mutations of *RHOA* p.Asn117Lys and p.Asn117Ile were specifically altered in human CTCLs (36); therefore, we selected these variants and investigated them in four canine TCL/NCL subtypes. Surprisingly, *RHOA*^N117I, N117K^ was found in all TCL subtypes with the highest frequency in EATCL (4/8, 50%), NCL (3/6, 50%), and ECTCL (10/21, 47%). Another SNP, *STAT3* p.Tyr640Phe, was recently discovered to be mutated in human NK-cell lymphoma and CTCL (19, 49). *STAT3* is a transcription factor that plays a key role in many cellular processes, including cell growth and apoptosis. Unlike in humans, *STAT3*^Y640F^ was rarely observed in canine TCLs/NCLs; it was found in four cases of CTCL (11%), one case of EATCL (12%), and one NCL (16%). Three SNP locations of missense (p.Lys78Arg), stop gained (p.Glu46Ter), and frameshift (p.Phe37LeufsTer35) variants of *PDCD1* were found to be loss-of-function mutations in human PTCLs (31). In addition, PD-1 deletions were related to a worse prognosis because they could revoke T-cell exhaustion and drive aggressive behavior in CTCL (19). In dogs, *PDCD1*^E46*, K78R, F37LX^ was detected in NCL (4/6, 66%) and NECTCL (5/15, 33%). Besides, the lack of complete clinical information in the current study concealed the association between the *PDCD1* mutation and prognosis. Thus, a prospective study of these SNPs affecting clinical stage, survival time, and disease progression is required to confirm their significance in prognosis.

Our study demonstrated that each canine patient with a specific subtype of TCL showed a different targeted SNP genotype. Hence, specific target therapy based on the SNP information may be advantageous in dogs with a certain type of lymphoma to increase treatment efficiency concurrent with chemotherapy. A study of CTCL in mice illustrated that methyltransferase inhibitors could restore *SATB1* function in the Sézary cell line (50), and the usage of this target drug possibly abrogates malignant expansion in CTCL dogs. A *STAT3* mutation could cause constitutive activation and might be a possible STAT3 inhibitor target. Napabucasin, a novel *STAT3* inhibitor, has shown promising effects in inducing intrinsic and extrinsic apoptosis and downregulating the expression of *STAT3* target genes against neoplastic B-cell lineages (51). Furthermore, napabucasin displayed a synergistic effect when administered with doxorubicin *in vitro* and *in vivo* experiments and may provide therapeutic implications in human B-cell lymphoma. Immunotherapy of PD-1 is an attractive cancer target. In human clinical trials, PD-1 blockage demonstrated substantial therapeutic activity to treat relapsed/refractory Hodgkin’s lymphoma as a single agent and advanced NK/TCL concurrent with chemotherapy (52, 53). The effectiveness of novel targeted drugs, particularly somatic SNPs, for treating canine lymphoma is necessitated in preclinical studies.

A small sample size of EATCL and NCL in the present study affected the estimated prevalence of each SNP between the two groups. Moreover, the lack of medical records for each patient hindered the valuable significance of SNPs and prognostic indicators. Another recently critical concern was FFPE-induced mutational artifacts, predominantly excessive T > C mutations, that may be affected in this study.

In conclusion, the MassARRAY platform revealed diverse mutational profiles, exhibiting significant variations across different TCL/NCL subtypes and their anatomical locations. Mutations in *SATB1*, *KIT*, *SEL1L*, and *TP53* are frequently observed. Somatic mutations, particularly in *TP53*, are detected, implying a potential disparity between germline and somatic mutational patterns, although this analysis requires further validation in a larger number of cases and their contribution if they are cancer-associated mutations in dogs.

## Data availability statement

The datasets presented in this study can be found in online repositories. The names of the repository/repositories and accession number(s) can be found in the article/Supplementary material.

## Ethics statement

The animal studies were approved by the Institutional Animal Care and Use Committee (IACUC), Chulalongkorn University Laboratory Animal Center. The studies were conducted in accordance with the local legislation and institutional requirements. Written informed consent was obtained from the owners for the participation of their animals in this study.

## Author contributions

SS: Formal analysis, Investigation, Methodology, Software, Validation, Visualization, Writing – original draft, Writing – review & editing. TK: Conceptualization, Investigation, Writing – review & editing. ST: Conceptualization, Supervision, Writing – review & editing. AR: Conceptualization, Funding acquisition, Methodology, Resources, Supervision, Writing – review & editing.

## Footnotes

^1^ https://www.ensembl.org
