# Supplementary material for: Single nucleotide polymorphism profiles of canine T-cell and null-cell lymphomas
Source: Front Vet Sci. 2024 Aug 7;11:1439706. doi: 10.3389/fvets.2024.1439706 (PMC11339873; doi:10.3389/fvets.2024.1439706)
Supplement: Supplementary file 1 [file Data_Sheet_1.pdf]

Supplementary Material

Single nucleotide polymorphism profiles of canine T-cell and null-cell lymphomas

Sirintra Sirivisoot, Tanit Kasantikul, Somporn Techangamsuwan, Anudep Rungsipipat*

*** Correspondence:** Anudep Rungsipipat: anudep.r@chula.ac.th

Supplementary Figures

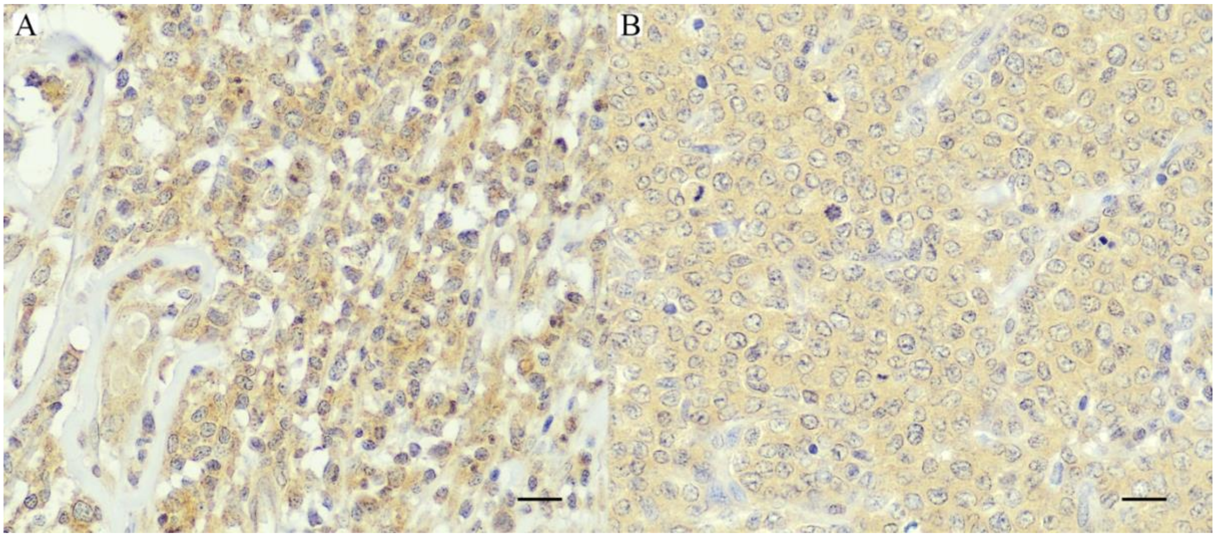

Supplementary Figure 1. Immunohistochemistry against programed death ligand 1 (PD-L1) in canine T-cell lymphoma. (A) The mutant *SATB1*^{Q420P} illustrates strong cytoplasmic intensity in 100% of neoplastic cells comparing to wild type (B). IHC. Bar = 10 μm

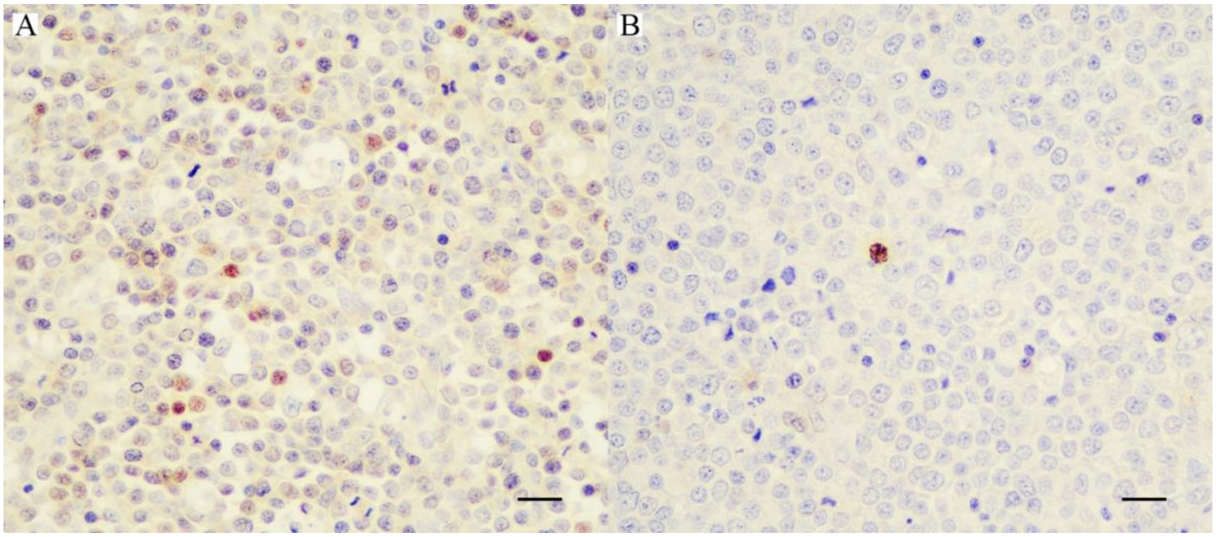

Supplementary Figure 2. Immunohistochemistry for p53 in canine null-cell lymphoma. (A) The wild-typed *TP53*^{I149N} presents heterogenous nucleus positive staining <10% of neoplastic cells, whereas the mutant shows absence expression <1% of nuclei stained (B). IHC. Bar = 10 μm.

Supplementary Table 1. The sequencing primers (5’ to 3’) of each SNP in this study.

SNP	Forward oligonucleotides	Reverse oligonucleotides
rs22299980	ACGTTGGATGGTCTGACCTATGGCCATTTC	ACGTTGGATGAATCCTGCAACCACACACTG
PTEN c.975C>T	ACGTTGGATGGGAGAAAAATATCGGTTGGC	ACGTTGGATGACGTGCAGATAATGACAAGG
HYALP6 c.1317A>G	ACGTTGGATGTGACGTTGTAAGAGGAGGTG	ACGTTGGATGGGAAGAATAAGGCTCCAAGG
LMNB1 c.1184C>T	ACGTTGGATGCCCATGTAGATTGAAACTCTC	ACGTTGGATGAACACTCCGACTTGAGGATG
MET c.3804C>G	ACGTTGGATGTTGGCGCCTGTTTTGTTGTG	ACGTTGGATGGTCAAGGTTGCTGATTTTGG
MVB12A c.361G>A	ACGTTGGATGTCTTGGTCTTCCCACTCAGC	ACGTTGGATGAACGCATGTGTGTGAAGCTG
MYC c.224C>T	ACGTTGGATGCCAGCGAGGATATCTGGAAG	ACGTTGGATGAAGGACGCGACTGCGACGTA
SATB1 c.1259A>C	ACGTTGGATGATAGCCCGAAGGTTTACCAG	ACGTTGGATGGGGCTTGCTTTCAGAAATCC
rs852661628	ACGTTGGATGCCACCATCCACTACAACTAC	ACGTTGGATGTCCAGGGTGATGATAGTGAG
TP53 c.1024C>T	ACGTTGGATGTCACTCACCTGGAGTGAGC	ACGTTGGATGTCTGAATGAAGCCTTGGAGC
TP53 c.311_312insA	ACGTTGGATGCTACAGTCTTCGGAGCTGTG	ACGTTGGATGAGTCTTCGTCTAGCCAGTTC
TP53 c.446T>A	ACGTTGGATGTGTCAGAGCAGCGTTCATGG	ACGTTGGATGAATACCTGCGTCCGCGCTA
TP53 c.640_641insT	ACGTTGGATGGTAGTTGTAGTGGATGGTGG	ACGTTGGATGTGAGGCTGATAGACTACAGG
PDCD1 c.136G>T	ACGTTGGATGTCTTTCCCCCGTCAGACTC	ACGTTGGATGCAGGTGAACGTGGCGTTCT
PDCD1 c.233A>G	ACGTTGGATGACAGCTTCGTGCTCAACTGG	ACGTTGGATGTCGATGCGGTCCTCCTGGAA
PDCD1 c.108_109insCT	ACGTTGGATGTCTTTCCCCCGTCAGACTC	ACGTTGGATGTGAACGTGGCGTTCTCTCC
POT1 c.850C>T	ACGTTGGATGTGAGGACCAGAAAATGGTAG	ACGTTGGATGTTTGCCCAAGAGCTGACAAG
POT1 c.927del	ACGTTGGATGTTTGCCCAAGAGCTGACAAG	ACGTTGGATGTGAGGACCAGAAAATGGTAG
*POT1*c.1747C>T	ACGTTGGATGTGTGGACCACTACAGATCAA	ACGTTGGATGAGACAGTCACTTGAAAGTGG
POT1 c.1928T>C	ACGTTGGATGTGAGATCTGGCCATGAAGAC	ACGTTGGATGCCCATAGTGATGAATTTTCCC
rs851689319	ACGTTGGATGTCCAGGTTTCCTTGCTACAG	ACGTTGGATGAGCTACATATCTGATTGTGC
TRAF3 c.906del	ACGTTGGATGTGCACAATCAGATATGTAGC	ACGTTGGATGCCTTTGATCCAGCCATAGTC
TRAF3 c.908dup	ACGTTGGATGCCTTTGATCCAGCCATAGTC	ACGTTGGATGTGCACAATCAGATATGTAGC
TRAF3 c.942_949dup	ACGTTGGATGTGCACAATCAGATATGTAGC	ACGTTGGATGCCTTTGATCCAGCCATAGTC
TRAF3 c.968_971del	ACGTTGGATGTCTTTGTCCAGCTCTTTCAG	ACGTTGGATGTGCTTTGCCTTTCTGATGCC
TRAF3 c.1652del	ACGTTGGATGTCCGAAGTATCCACTATGAC	ACGTTGGATGTGTTCGTGGCTCAAACTGTG
TRAF3 c.1434_1445del	ACGTTGGATGTTCTGCTTGAATGGCCAAGG	ACGTTGGATGGACGCACTTGTCGCTGTTTT
TRAF3 c.1591_1592insTC	ACGTTGGATGCCAACAGCAGCAGCTTCAAG	ACGTTGGATGAGTTTGAGCCACGAACACTG
TRAF3 c.1339del	ACGTTGGATGCCCTGGCACACATCTTATAG	ACGTTGGATGACCCTGTCTCTTTACAGCC
TRAF3 c.1195del	ACGTTGGATGCAGATGCTGAGCGTCCATGA	ACGTTGGATGAAATCAGCACGCCGTTGTAG
STAT3 c.1919A>T	ACGTTGGATGGACATGTTGTTCAGCTGTTG	ACGTTGGATGCCCTTACAATATCCCATGAC
RHOA c.350A>T	ACGTTGGATGGAAGTCAAGCACTTCTGTCC	ACGTTGGATGCTTGTGTGCTCATCATTCCG
RHOA c.351C>A/G	ACGTTGGATGCTTGTGTGCTCATCATTCCG	ACGTTGGATGGAAGTCAAGCACTTCTGTCC
SPAM1 c.1445A>C/G/T	ACGTTGGATGAAACTCAGTACCCAGTGGTC	ACGTTGGATGGGCAGAGAAGATATTGCTGG
FLT3 c.10+1601A>C	ACGTTGGATGCCTCAGAAATGGGAAATGGG	ACGTTGGATGCTCTGTCTTCCCTTCATTGC
FLT3 c.10+1830A>G	ACGTTGGATGCAAATGGGAGTTTCCCAGAG	ACGTTGGATGGAGATTTCCCAACAGAAAGG
FLT3 c.10+13857G>C	ACGTTGGATGGTAAGGCCGCTATTTTGCAC	ACGTTGGATGACATGTACTACAGCGGTTGG
ZNHIT6 c.-14G>C	ACGTTGGATGACAGCAATATCCATCAGCCC	ACGTTGGATGTTCCTTTTGGCGTGGACAAG
DIO2 c.-128-3748T>G	ACGTTGGATGCCTGCTAGCTAGCCAAAATC	ACGTTGGATGGTGAGGGAAAAGGGTACAAC
SEL1L c.2040+200C>G	ACGTTGGATGCTGTTTTTGGCTTACTATCC	ACGTTGGATGCTTGTCATTGGACACATGCC
SEL1L c.1248+56G>C	ACGTTGGATGGAGGTGTTTGCATAATAAGC	ACGTTGGATGACCACAAGAGACAATTATCC
SEL1L c.777+1097T>C	ACGTTGGATGCCACACCATCAATAAACCTG	ACGTTGGATGAGATTGACACCTGGGTTTAG
SNORD3A	ACGTTGGATGAATCATGAAAGAAGCCGTGC	ACGTTGGATGGGCGTAAGAGAGCACTAAAC
ENSCAFG00000053717	ACGTTGGATGGCTCCTCTCACAAACACTTC	ACGTTGGATGCAAACATCCATGGACGATGA
